# Preliminary assessment of the computer‐based *Taenia solium* educational program ‘The Vicious Worm’ on knowledge uptake in primary school students in rural areas in eastern Zambia

**DOI:** 10.1111/tmi.13029

**Published:** 2018-01-29

**Authors:** Emma C. Hobbs, Kabemba Evans Mwape, Inge Van Damme, Dirk Berkvens, Gideon Zulu, Moses Mambwe, Mwelwa Chembensofu, Isaac Khozozo Phiri, Maxwell Masuku, Emmanuel Bottieau, Brecht Devleesschauwer, Niko Speybroeck, Angela Colston, Pierre Dorny, Arve Lee Willingham, Sarah Gabriël

**Affiliations:** ^1^ One Health Center for Zoonoses and Tropical Veterinary Medicine Ross University School of Veterinary Medicine Basseterre St Kitts, West Indies; ^2^ Institute of Tropical Medicine Antwerp Belgium; ^3^ Faculty of Veterinary Medicine Ghent University Merelbeke Belgium; ^4^ School of Veterinary Medicine University of Zambia Lusaka Zambia; ^5^ Ministry of Health Government of the Republic of Zambia Lusaka Zambia; ^6^ Scientific Institute of Public Health Brussels Belgium; ^7^ Institute of Health and Society Université Catholique de Louvain Brussels Belgium; ^8^ Global Alliance for Livestock Veterinary Medicines (GALVmed) Nairobi Kenya

**Keywords:** disease control, health education, One Health, *Taenia solium*, taeniasis/cysticercosis, Zambia, contrôle des maladies, éducation à la santé, One Health, *Tænia solium*, téniase/cysticercose, Zambie

## Abstract

**Objective:**

The zoonotic helminth *Taenia solium* is endemic in Zambia, causing human (taeniasis and (neuro)cysticercosis) and pig (porcine cysticercosis) diseases with high health, social and economic burdens. We aimed to evaluate the impact of a health educational program intended to lead to powerful and cumulative improvements in knowledge, attitudes and practices that decrease parasite transmission and disease occurrence.

**Methods:**

Half‐day health education workshops were conducted in three primary schools in the highly endemic Eastern Province of Zambia, using the computer‐based *T. solium* educational program ‘The Vicious Worm’. Questionnaires were administered before and after the educational component to determine the program's impact on knowledge uptake in primary school students.

**Results:**

In total, 99 students participated: 38 males and 61 females, with a median age of 14 years (range 10–18 years). Baseline general knowledge of *T. solium*, including awareness of the different human and pig disease states, and disease diagnosis, treatment and prevention, was quite high (average score 62%) and consistent across all three study areas. Participants’ knowledge had significantly increased after the educational component, particularly regarding parasite transmission and disease prevention.

**Conclusion:**

Preliminary assessment of ‘The Vicious Worm’ indicates it is an effective tool for the short‐term *T. solium* education of primary school students in Zambia. Follow‐up studies are planned to assess the longer term impact of the program on knowledge uptake in the study neighbourhoods. Inclusion of tailored ‘The Vicious Worm’ educational workshops should be considered in integrated cysticercosis control programs in endemic areas of sub‐Saharan Africa.

## Abstract (fr)

**Objectif:**

L'helminthe zoonotique *Tænia solium* est endémique en Zambie, causant des maladies humaines (téniase et (neuro) cysticercose) et porcines (cysticercose porcine) avec une charge sanitaire, sociale et économique élevée. Nous voulions évaluer l'impact d'un programme éducatif sur la santé destiné à apporter des améliorations importantes et cumulatives dans les connaissances, les attitudes et les pratiques qui réduisent la transmission des parasites et la survenue de maladies.

**Méthodes:**

Des ateliers d'une demi‐journée sur l’éducation à la santé ont été organisés dans trois écoles primaires de la province de l'Est de la Zambie, hautement endémique, à l'aide du programme informatisé intitulé *T. solium* «Le Vers Pervers». Des questionnaires ont été administrés avant et après le volet éducatif afin de déterminer l'impact du programme sur l'assimilation des connaissances chez les élèves du primaire.

**Résultats:**

Au total, 99 élèves ont participé: 38 garçons et 61 filles, avec un âge médian de 14 ans (intervalle: 10‐18 ans). La connaissance générale de base sur le *T. solium*, y compris la connaissance des différents stades pathologiques humains et porcins, et le diagnostic, le traitement et la prévention de la maladie, était assez élevée (score moyen de 62%) et constante dans les trois zones d’étude. Les connaissances des participants ont considérablement augmenté après le volet éducatif, en particulier, en ce qui concerne la transmission du parasite et la prévention de la maladie.

**Conclusion:**

L’évaluation préliminaire du «Le Vers Pervers» indique qu'il s'agit d'un outil efficace pour l’éducation à court terme sur le *T. solium* des élèves du primaire en Zambie. L'inclusion d'ateliers pédagogiques spécifiques du «Le Ver Pervers» devrait être envisagée dans les programmes intégrés de lutte contre la cysticercose dans les zones endémiques de l'Afrique subsaharienne.

## Introduction

The taeniasis/cysticercosis complex is caused by the zoonotic helminth *Taenia solium*, or ‘pork tapeworm’. Infections in humans (taeniasis, TS, and (neuro)cysticercosis, N/CC) and pigs (porcine cysticercosis, PCC) cause significant health and socio‐economic burdens in endemic countries throughout sub‐Saharan Africa, Latin America and southern Asia; the parasite is estimated to infect 53 million people and cause 28 000 human deaths every year 1, 2.

Although theoretically eradicable 3, high levels of active transmission persist in many developing countries, including Zambia, where pigs are free‐ranging, outdoor defecation is practiced, health education is rudimentary and meat inspection is limited or absent. Effective *T. solium* control tools include anthelmintics and pig vaccines, and non‐specific measures such as improvements to sanitation and the corralling of pigs. Several studies trialling one or more control strategies in endemic communities successfully reduced disease prevalence and incidence in the short term 4, 5, 6, 7, 8, 9, 10; however, sustained long‐term interruption of parasite transmission has not been achieved to date.

Given the complexity of the *T. solium* life cycle and the unintuitive links between open defecation, free‐ranging pigs and epilepsy, poorly educated communities may not see the point in adhering to externally imposed control strategies, such as confining pigs or using latrines. Health education interventions specifically targeting *T. solium* have been trialled in some countries including Tanzania, Kenya, India and Mexico; results showed increased knowledge about the parasite and its life cycle, and in some cases, decreased prevalence of TS and/or CC in humans and pigs was also recorded 11, 12, 13, 14. Focussing educational messages on school students can enable them to be ‘health change agents’ capable of disseminating messages throughout their communities 11, 15, 16.

‘The Vicious Worm’ is a freely downloadable computer‐based educational program developed by researchers from the University of Copenhagen (https://theviciousworm.sites.ku.dk). It uses cartoons, videos and quizzes to provide comprehensive information about *T. solium* in a fun and interactive way, set within an illustrated sub‐Saharan African context. Village, Town and City sections present varying levels of complexity, allowing delivery of specific messages to different audiences. Testing of the program on a group of Tanzanian medical and agricultural professionals revealed significant improvements in study subjects’ knowledge, regardless of gender, educational level, expertise with computers or prior familiarity with the parasite 17. Computer‐based tools have the advantages of providing standardised educational messages, reduce training costs, can be widely disseminated and can be updated as needed 18.

This study aimed to evaluate the effects of ‘The Vicious Worm’ on *T. solium‐*associated knowledge uptake in primary school students from the highly endemic Eastern Province of Zambia.

## Materials and methods

### Study area

**Figure 1:**
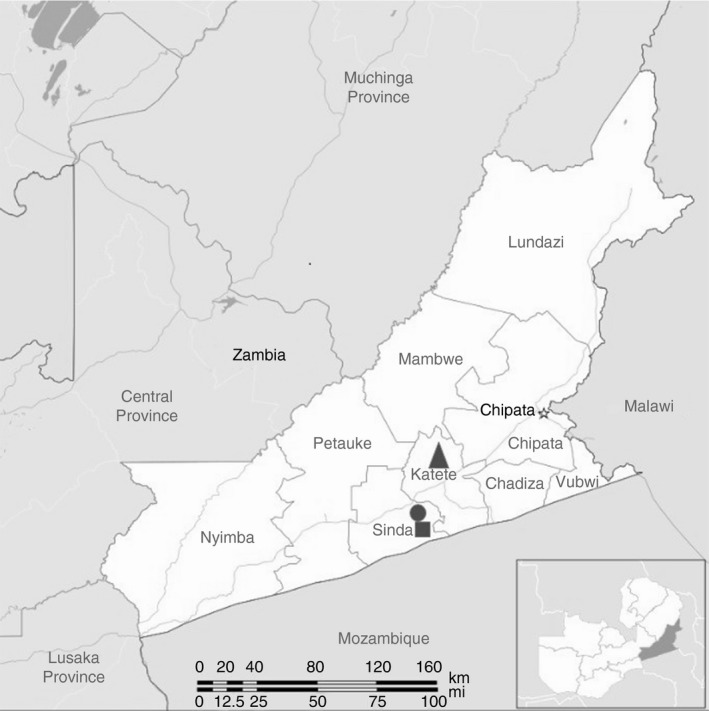
Map of Zambia showing the study areas in the Eastern Province: triangle = Nyembe neighbourhood community; circle = Herode neighbourhood community; square = Chimvira neighbourhood community.

The study was conducted in the Nyembe (Katete District), Chimvira and Herode (Sinda District) neighbourhood communities in the Eastern Province of Zambia (Figure 1). Approximately 98% of the region's estimated 66 000 pigs are reared under small‐scale ‘backyard’ conditions where free‐ranging and scavenging of human faeces are common 19. Open defecation is regularly practiced in the study districts despite over 90% of households having latrines (personal observations).

The region is hyperendemic for *T. solium* infections, with active prevalence of TS 6.3–11.9% (by copro‐Ag ELISA), of active human CC of 5.8–14.5% (based on circulating antigen detection, AgELISA), and of PCC up to 64% 20, 21, 22. The adjusted epilepsy prevalence in rural Zambia was estimated as 12.5/1000 inhabitants 23, and one recent study conducted in Katete‐identified NCC lesions in 57% of people with epilepsy, making it the most important cause of acquired epilepsy in this area 24.

### Study design

CYSTISTOP is a prospective, large‐scale community‐based intervention study conducted in Katete and Sinda Districts in the Eastern Province of Zambia. Village‐based educational sessions, incorporating generic and *T. solium*‐specific health messages, are a component of each of the three study arms, and are conducted at four‐ (Nyembe) or twelve‐monthly (Chimvira and Herode) intervals in conjunction with other project activities.

### Educational workshops

Students from grades three to six at the Nyembe (Nyembe), Kondwelani (Chimvira) and Gunda (Herode) primary schools were invited to attend a half‐day educational workshop in July 2016 (Nyembe) or November 2016 (Chimvira and Herode).

The workshop consisted of (i) a brief introductory session, (ii) a fun practice quiz, (iii) a ‘pre’ questionnaire, (iv) ‘The Vicious Worm’ educational component, comprising the introductory and village program sections, and (v) a ‘post’ questionnaire (a rearranged version of the ‘pre’ questionnaire). A generator‐powered laptop and projector were set up in an empty classroom for delivery of the workshops, which were conducted in Chewa.

### Questionnaire design

We simplified the original ‘Vicious Worm’ assessment questionnaire 25 and adapted some terminology for the Zambian context. The resulting questionnaire (QS1) (Supporting Information), used in the first (Nyembe) workshop, had 24 multiple‐choice questions, each with five to seven possible answers, and some with more than one answer that could be deemed correct. QS1 took approximately 90 min to complete, which proved too long and complicated for the students, who became visibly restless halfway through each round. Further revisions produced QS2, having 15 multiple‐choice questions, each with four answer options, and only one correct answer per question. QS2 was used in the next two (Chimvira and Herode) workshops and took approximately 35 min to complete.

Both questionnaires were designed to test knowledge of TS, human N/CC and PCC including the linkages between the disease states, and methods of transmission, diagnosis, and prevention. Questions were grouped into eight (QS1, as per Ertel *et al*. (2015)) or three (QS2) categories (Table 1).

**Table 1:** Categories in each of the questionnaires (QS) used in the workshops

Cat no.	Category	No. of questions
Questionnaire 1 (QS1)		24
n/a	Have you ever heard of *masese* a?	1
1	Acquisition & transmission of *T. solium* infections	3
2	Acquisition of NCC	2
3	TS in general	3
4	NCC in general	3
5	PCC diagnosis	4
6	PCC treatment	2
7	Relationship between PCC/TS/NCC	3
8	Prevention of PCC/TS/NCC	3
Questionnaire 2 (QS2)		15
1	General knowledge	5
2	Transmission	5
3	Prevention	5

NCC, neurocysticercosis; TS, taeniasis; PCC, porcine cysticercosis.

a ’Masese’ is the local (Chewa) word for porcine cysticercosis.

### Questionnaire delivery

Questions and answer choices were read aloud and repeated at least once for clarity. Each student then submitted his/her answer (‘voted’) using a Bluetooth‐connected TurningPoint^©^ clicker device. Once everyone had voted, the group proceeded onto the next question. During the ‘post’ round, after voting was closed for each question, the correct answer was revealed, and discussed with the group to address any remaining misconceptions.

### Data management and statistics

The differences in the two questionnaires prevented direct comparison of response data, so were analysed separately. Technical problems in Nyembe prevented collection of individual response data from QS1. Response data from the Chimvira and Herode groups were combined for analysis of QS2.

Group (QS1 and QS2) and individual (QS2) responses to each questionnaire session were exported into an Excel (Microsoft Corporation, 2010) spreadsheet for descriptive statistics. Questions were assessed individually and by category. Some questions in QS1 had more than one correct answer; selection of any one of these answers resulted in a ‘correct’ outcome. Data were exported to STATA 14.1 (StataCorp) for statistical analysis, and logistic regressions were performed to compare ‘pre’ and ‘post’ results.

The individual response data obtained from QS2 allowed further analysis: correct and incorrect answers were assigned numerical values (1/0) and the results were summed for each individual. The total was used as outcome variable. The difference between ‘pre’ and ‘post’ questionnaire results were also compared using Poisson regressions, controlling for the total number of questions (introduced as offset). Category data were similarly compared. Univariate and multivariable Poisson regressions were also used to assess the effect of different explanatory variables (study neighbourhood (Herode *vs*. Chimvira), gender (male *vs*. female), and age (≤15 *vs*. >15 years)) and their two‐way interactions on the ‘pre’ QS2 questionnaire data, controlling for exposure (being the number of questions in total, or within a category, as applicable). ‘Improvement’ was defined as the number of questions that changed from incorrect (‘pre’) to correct (‘post’). An individual's ‘potential for improvement’ was defined as the difference between the total number of questions, and the number of that individual's correct answers during the ‘pre’ round. Improvement was calculated to evaluate the effect of the different explanatory variables on the knowledge uptake after the Vicious Worm demonstration. The ‘potential for improvement’ was included as exposure. To evaluate if the improvement was different from zero, a one‐sample *t*‐test was performed.

### Ethical considerations

This study was conducted within the ongoing CYSTISTOP project. Ethical clearance was obtained from the University of Zambia Biomedical Research Ethics Committee (004‐09‐15) and the Ethical Committee of the University of Antwerp, Belgium (B300201628043, EC UZA16/8/73). The study was introduced and explained to all project participants, both in village group settings and within individual households, prior to each field visit. Written informed consent to participate in the workshop, voluntarily provided by a parent or guardian, was obtained for each student, and the students’ attendance at the educational workshops was voluntary. The workshops took place outside of normal school hours. There was no incentive for participation, but light refreshments were provided.

## Results

A total of 99 students participated in the three workshops: 38 males and 61 females. Ages ranged from 10 to 18 years, with a median of 14 years.

### Baseline knowledge (‘pre’ questionnaire): Results by question

Regarding QS1 data, correct answers ranged from 18% to 100%, with an average score of 62% (see Table 2). Of the 24 questions, 8 (33%) were answered correctly by at least 75% of the group, and 2 (8.3%) were answered correctly by 90% or more of the group. The whole group correctly answered, ‘What are the symptoms of NCC?’ Five questions (21%) were answered correctly by <50% of the group; ‘What should be done with a slaughtered pig that has CC?’ had the lowest percentage (18%) of correct answers.

**Table 2:** Average results from ‘pre’ and ‘post’ Vicious Worm questionnaires (QS1), *n* = 40

Question	Correct during ‘pre’ QS1 (%)	Correct during ‘post’ QS1 (%)	Knowledge uptake (%)	*P*‐value for difference
Have you ever heard about *masese* a?	70.0	75.0	5.0	NS
Category 1: Acquisition and transmission of *T. solium* infections	65.3	85.0	19.7	<0.001
How can a pig become infected with PCC?	78.0	90.0	12.0	NS
How do people get *T. solium* TW infection?	50.0	85.0	35.0	0.001
A person infected with a *T. solium* TW will shed many eggs through…?	68.0	80.0	12.0	NS
Category 2: Acquisition of NCC	26.5	20.5	−6.0	NS
A person with N/CC might have got the infection by…?	25.0	13.0	−12.0	NS
A person with NCC can transmit the disease to others through…?	28.0	28.0	0.0	NS
Category 3: TS in general	74.3	85.3	11.0	0.039
What is human TW infection/TS?	65.0	80.0	15.0	NS
How can human TW infection/TS be diagnosed?	75.0	88.0	13.0	NS
How can TS be treated?	83.0	88.0	5.0	NS
Category 4: NCC in general	69.7	87.0	17.3	0.001
What is human NCC?	35.0	73.0	38.0	0.001
What are the symptoms of NCC?	100.0	98.0	−2.0	NS
What should a person who experiences seizures/chronic headache do?	73.0	90.0	17.0	NS
Category 5: PCC diagnosis	68.8	83.8	15.0	0.002
What is PCC?	75.0	100.0	25.0	0.023
What does PCC look like?	60.0	85.0	25.0	0.015
How can you test for PCC in a live pig?	60.0	90.0	30.0	0.004
How can PCC be diagnosed in a slaughtered pig?	80.0	60.0	−20.0	NS
Category 6: PCC treatment	35.0	41.5	6.5	NS
What should ideally be done with a live pig that has PCC?	52.0	75.0	23.0	0.023
What should be done with a slaughtered pig that has PCC?	18.0	8.0	−10.0	NS
Category 7: Relationship between PCC/TS/NCC	61.0	67.7	6.7	NS
Is PCC a problem for human health?	88.0	88.0	0.0	NS
Are PCC and human TW related?	65.0	55.0	−10.0	NS
What problems can an adult *T. solium* TW cause?	30.0	60.0	30.0	0.008
Category 8: Prevention of PCC/TS/NCC	70.3	85.3	15.0	0.004
How can you prevent pigs getting PCC?	93.0	98.0	5.0	NS
How can you prevent human TW infections?	52.0	70.0	18.0	NS
How can human CC/NCC be prevented?	66.0	88.0	22.0	0.002
OVERALL QUESTIONNAIRE AVERAGES	62.0	73.5	11.5	<0.001

CC, cysticercosis; TW, tapeworm; TS, taeniasis; PCC, porcine cysticercosis; NCC, neurocysticercosis; NS, not significant.

a ’Masese’ is the local (Chewa) word for porcine cysticercosis.

Regarding QS2 data, the average score was 62%, ranging from 34% to 100% (Table 3). Five questions (33%) were answered correctly by 75% or more of the group, one of which was correctly answered by all. Six questions (40%) were poorly answered, with <50% of the group answering correctly.

**Table 3:** Average results from ‘pre’ and ‘post’ Vicious Worm questionnaires (QS2), *n* = 59

Question	Correct during ‘pre’ QS2 (%)	Correct during ‘post’ QS2 (%)	Knowledge uptake (%)	*P*‐value for difference
Category 1: General knowledge	68.1	79.0	10.8	0.125
Have you ever heard of *masese* a?	76.3	96.6	20.3	ND
What is PCC?	84.7	100.0	15.3	ND
What does PCC look like in pigs?	74.6	91.5	16.9	ND
What is human tapeworm/TS?	59.3	72.9	13.6	ND
What are the symptoms of NCC?	59.3	93.2	33.9	ND
Category 2: Transmission	50.8	80.7	29.8	0.000
How can a pig become infected with PCC?	33.9	91.5	57.6	ND
How do people get TS?	78.0	100.0	22.0	ND
How does a person with TS shed eggs into the environment?	49.2	86.4	37.3	ND
How do people get CC?	33.9	32.2	−1.7	ND
Can people with NCC transmit it to others?	45.8	33.9	−11.9	ND
Category 3: Prevention	65.8	86.4	20.7	0.004
Can you eat the meat of a slaughtered pig with PCC?	49.2	89.8	40.7	ND
Can you prevent pigs getting PCC?	44.1	86.4	42.4	ND
How can TS be treated?	100.0	98.3	−1.7	ND
How can you prevent TS?	81.4	96.6	15.3	ND
How can CC and NCC be prevented?	54.2	61.0	6.8	ND
OVERALL QUESTIONNAIRE AVERAGES	61.6	82.0	20.5	0.000

PCC, porcine cysticercosis; TW, tapeworm; TS, taeniasis; CC, cysticercosis; NCC, neurocysticercosis; ND, calculation was not made (as significance was already determined by category).

a ’Masese’ is the local (Chewa) word for porcine cysticercosis.

The ‘potential for improvement’ calculations revealed that on average, 5.8 questions (of 15 in total) were answered incorrectly by respondents: 5.9 for males and 5.6 for females.

### Results by category

Regarding QS1 data, the category scores ranged from 27% to 74%, with six of the eight categories (75%) answered correctly by between at half the group. The least understood category was ‘Acquisition of NCC’.

Regarding QS2 data, the three categories were answered correctly by between 51% and 66% of the group; averages were 68% for ‘General knowledge’, 51% for ‘Transmission’ and 66% for ‘Prevention’.

### End knowledge (‘post’ questionnaire): Results by question

On QS1 data, correct answers ranged from 8% to 100%, with an average score of 73%. Eight questions (33%) were answered correctly by at least 75% of the group, and two (8.3%) by 90% or more. One question was answered correctly by all. Five questions (21%) were answered correctly by less than half the group. The question ‘What should you do with a slaughtered pig with PCC?’ had the lowest percentage of correct answers, with most respondents (83%) selecting ‘Destroy the pig’ as the suitable answer.

On QS2 data, the average score for this round was 82%, with 13 of the 15 questions (87%) answered correctly by at least half of the group. Eleven questions (73%) were correctly answered by at least three‐quarters of the group, and two questions (13%) were answered correctly by the whole group. The two least successfully answered questions were related to N/CC transmission.

### Results by category

Category scores for QS1 data ranged from 20% to 87%, with an average of 84%. Five categories (63%) were answered correctly by at least 80% of the group. The categories ‘PCC treatment’ and ‘Acquisition of NCC’ were again poorly answered this round, with scores of 42% and 21%, respectively. Regarding QS2 data, these three categories averaged 82% this round. Individual category scores ranged from 79% to 86%, with ‘Prevention’ the most successfully answered.

### Knowledge uptake: Change in knowledge uptake by question

In total, 17 (71%) of the 24 questions on QS1 data were answered more successfully during the post‐round, of which eight were statistically significant (*P* < 0.05). Increases of 10% or more were seen for 14 (82%) questions, and six (35%) increased by at least 25%.

The average proportion of correct answers for QS1 increased by 17.5%. The number of questions that had been correctly answered by at least 75% of the group doubled, and those answered correctly by at least 90% of the group tripled, from two (8.3%) to six (25%). In both rounds, one question was answered correctly by the entire group, although it was a different question each time. No change was seen for two (8%) of the questions; however for five of the questions (21%), knowledge uptake fell by 2–20%.

The average score on QS2 data increased from 62% (‘pre’) to 82% (‘post’) (*P* < 0.001). Increases were seen in 12 of the 15 questions (80%), ranging between 7% and 58%. Correct answers to the question ‘How can a pig become infected with PCC?’ increased the most, from 34% (‘pre’) to 92% (‘post’). Correct answers to 11 questions (65%) increased by at least 10%, and to five questions (29%) by 25% or more.

Three questions (27%) saw knowledge deterioration. Correct answers to two decreased only slightly; one was still very well answered (100% to 98%), whereas the other (‘How do people get CC?’) was poorly understood during both rounds, with most respondents selecting ‘By eating undercooked pork infected with *T. solium*’ as the suitable answer. Correct answers to the third question, ‘Can a person with NCC transmit the disease to others?’, fell from 46% in the ‘pre’ round to 34% during the ‘post’ round, with the majority of the group selecting ‘Yes, by coughing or sneezing’ during each time.

On average, 4.0 questions improved from being answered incorrectly in the ‘pre’ questionnaire round to correct in the ‘post’ round (4.3 for males, 3.6 for females). No significant differences were found between villages (*P* = 0.503), gender (*P* = 0.446) or age (*P* = 0.926).

### Change in knowledge uptake by category

Increased knowledge was seen in seven of the eight QS1 categories (88%), with five (71%) increasing by 10% or more (*P* < 0.05). The highest increase was seen for the ‘Acquisition and transmission of *T. solium* infections’ category, from 65% (‘pre’) to 85% (‘post’).

The ‘Acquisition of NCC’ category decreased from 27% to 21%; in both ‘pre’ and ‘post’ rounds, most respondents selected ‘stool’ and ‘eating undercooked infected pork infected with *Taenia solium*’ in response to the questions, ‘A person with NCC can transmit it to others via…?’ and ‘A person with N/CC might have got the infection by…?’, respectively.

Regarding QS2 data, increases of at least 10% were seen in all three categories. The ‘Transmission’ category demonstrated the highest change with a 30% increase (*P* < 0.001), whereas the ‘Prevention’ category increased by 19% (*P* = 0.004). Improvement for QS2 was significant both overall and for each of the categories (*P* < 0.001).

## Discussion

This study was the first to assess the impact of the computer‐based cysticercosis educational tool ‘The Vicious Worm’ on knowledge of *T. solium*‐associated diseases in school students in Zambia. Baseline knowledge of *T. solium* was quite high (average score 62%) and consistent across all three study areas. Significant increases were seen immediately after the educational workshops, both overall, and more particularly relating to transmission and prevention, and were independent of gender, age or study neighbourhood.

The increased knowledge seen in seven of eight (88%, QS1) and three of three (100%, QS2) questionnaire categories indicates that overall, the key concepts for parasite control (including hand washing, proper cooking of pork, confining pigs and use of latrines) were better understood by the students after the educational workshops. However, some aspects of the parasite's life cycle were not well understood. Regarding management of infected pigs, many respondents recommended destruction of live pigs and/or pig carcasses during both ‘pre’ and ‘post’ questionnaire rounds. Properly cooking/boiling the meat to destroy the parasite was technically the ‘correct’ answer; however, discussions with villagers revealed that in practice, slaughtering infected pigs (and burying the carcass) is in fact their most reliable method of ensuring infected meat is not consumed or sold. Therefore, although these responses were marked as incorrect during the workshops, it appears that in reality, the villagers do understand the role of infected pigs in disease transmission and are taking appropriate steps to prevent this from occurring.

Acquisition of NCC was and remained poorly understood, with many respondents selecting coughing or sneezing (58%, QS2), or stool (48%, QS1) as transmission methods. Another sustained misconception was that N/CC develops after consuming undercooked infected pork (63% of respondents, QS2). Confusion regarding NCC transmission and the human–pig disease linkages was also described by Ertel *et al*. (2015) after trialling ‘The Vicious Worm’ educational workshops with adult agricultural and medical professionals in Tanzania.^1^ Similar results were also obtained from educational *T. solium* trials of varying designs in endemic community groups in Mexico, India, Tanzania and Kenya 11, 12, 13, 14, 16. This misperception likely reflects the complexity of the parasite's life cycle and the unintuitive links between human and pig disease states. Sarti *et al*. 13 suggested that *T. solium* education is more successful when describing obvious or tangible aspects of the life cycle (e.g. visible cysts in infected pigs) as compared to more abstract concepts (such as ingestion of invisible eggs leading to human N/CC). It may be advisable to simplify future workshops in future to prevent confusion, and instead focus on the key methods for prevention of parasite transmission. Indeed, the *T. solium* life cycle could be omitted altogether. Providing simple leaflets or other take‐home materials may also encourage clear, unambiguous communication of these messages to family and community members.

This study has limitations. The differences in questionnaires used and the unavailability of individuals’ response data from the Nyembe workshop prevented a comprehensive comparison of knowledge and knowledge uptake across the three study groups on the individual level. This was, however, deemed preferable to risking further concentration fatigue in study participants by persisting with QS1. Another limitation was the relatively small sample size (three schools, 99 students). However, the significant differences detected in the ‘pre’ and ‘post’ comparison data from each of the two QS indicate that the study design was of value.

Of course, this is not the only tool that may be effective for *T. solium* education. Also, the extent of knowledge transfer from the school students to their families and communities is unknown and difficult to quantify. Furthermore, increases in knowledge and awareness of *T. solium* do not necessarily lead to changes in transmission‐related behaviours. Indeed, some studies have shown that behaviours such as consuming raw pork, non‐use of latrines and allowing pigs to roam free may continue in some areas despite individuals knowing that they put themselves and their families at risk of infection 12, 13. These behaviours may have cultural, economical and/or practical drivers which outweigh the perceived threat of infection, including cultural taboos associated with sharing latrines with certain members of the opposite sex 26, religious significance of raw pork consumption in rituals and ceremonies 27, or inability to afford supplemental feedstuffs for confined pigs 28. Further research and development of culturally specific health educational campaigns will maximise their effectiveness.

Logistically, taking ‘The Vicious Worm’ out to rural primary schools proved fairly straightforward: delivery of the educational component required only a laptop, projector and small generator. The students were clearly engaged by the program's animations that clearly depicted their own sub‐Saharan African village settings. Further tailoring of the program by the workshop presenter, by assigning common local surnames to the different story characters, also proved popular and would be encouraged in future workshops.

## Conclusion

This preliminary assessment demonstrates that ‘The Vicious Worm’ is an effective and adaptable cysticercosis educational tool for primary school students in rural Zambia. Despite some aspects of the *T. solium* life cycle remaining imperfectly understood, key messages for prevention of parasite transmission were better understood by the students after the workshops. Follow‐up workshops are planned to assess the long‐term impact of the program on knowledge retention in the study areas. ‘The Vicious Worm’ should be considered for inclusion with carefully targeted interventional and/or educational campaigns in *T. solium* endemic areas in sub‐Saharan Africa in future.

## Supporting information


**Appendix S1.** Statistical calculations for QS2.
**Appendix S2.** Questionnaires.

## Note

1 It should be noted that the format of the workshops in this study differed from the Tanzanian workshops: our study participants were shown ‘The Vicious Worm’ as a group, rather than interacting with the program on individual laptops.
