# Supplementary material for: Preliminary assessment of the computer‐based Taenia solium educational program ‘The Vicious Worm’ on knowledge uptake in primary school students in rural areas in eastern Zambia
Source: Trop Med Int Health. 2018 Jan 29;23(3):306–14. doi: 10.1111/tmi.13029 (PMC5888122; doi:10.1111/tmi.13029)
Supplement: Supplementary file 1 — Appendix S1. Statistical calculations for QS2. Appendix S2. Questionnaires. [file TMI-23-306-s001.docx]

**Supplementary Material 1: Statistical calculations for QS2**

Supp. Table 1: Significance of explanatory variables for ‘pre’ and ‘post’ questionnaires (QS2). Factors analysed were village (Chimvira vs. Herode), gender (male vs. female), and age group (15 years of age and younger vs. 16 years and older [age range 10-18 years])

| **Explanatory variable** | | **p-value** |
| --- | --- | --- |
| ‘Pre’ questionnaire | |  |
|  | Category 1  Village  Gender  Age group  Gender + age group | 0.952  0.712  0.622  0.377 |
|  | Category 2  Village  Gender  Age group | 0.522  0.646  0.903 |
|  | Category 3  Village  Gender  Gender + age group | 0.943  0.992  0.925 |

**Supplementary Material 2: Questionnaires**

**2a. Questionnaire 1 (QS1)**

1. Have you ever heard about *masese/mase/mushokwe*?
2. No
3. Yes, as a human disease
4. Yes, as a disease in pigs
5. Yes, as a disease in both humans and pigs
6. Do not know
7. What is porcine cysticercosis, or The Vicious Worm in pigs?
8. Disease with white cysts caused by the tapeworm Taenia solium
9. Disease with white cysts caused by maize bran
10. Wounds in the skin caused by thorns
11. Do not know
12. What does porcine cysticercosis look like?


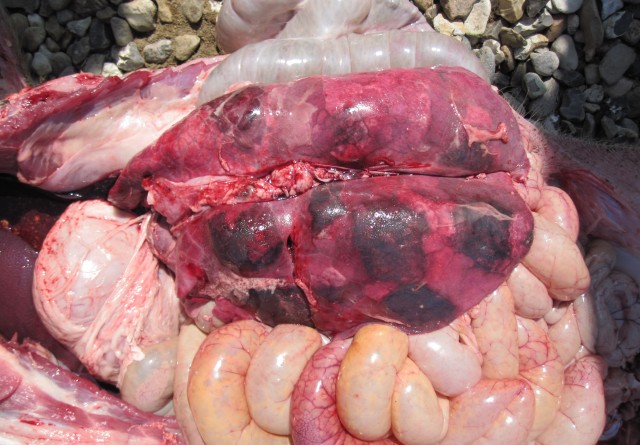

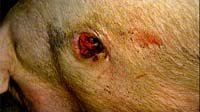


Picture A: black areas on the lungs Picture B: wounds in the skin


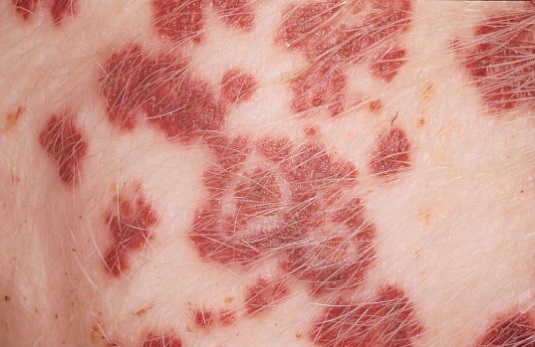


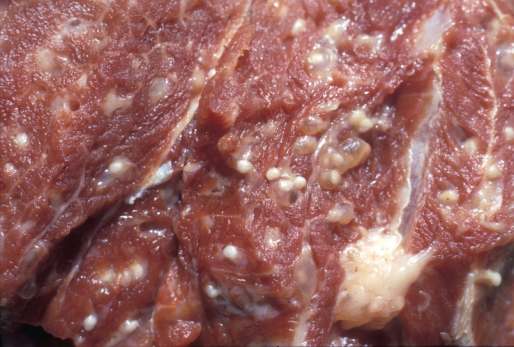


Picture C: White cysts in the muscles and organs Picture D: Red circular marks in the skin


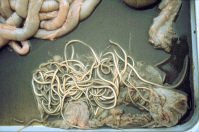


Picture E: Worms in the intestine

1. How can a pig become infected with porcine cysticercosis?
2. From dirt getting into wounds
3. From eating human stool
4. From eating mouldy maize bran or rice
5. From being mated with an infected pig
6. It was born infected
7. Do not know
8. How can you test porcine cysticercosis in a live pig?
9. By examination of the tongue
10. By examination of the feet
11. By examination of the ears
12. By examination of the snout
13. You cannot test it
14. Do not know
15. How can porcine cysticercosis be diagnosed in a slaughtered pig?
16. By inspecting the stomach and intestines for worms
17. By inspecting the skin for red marks
18. By inspecting the lungs for black areas
19. By inspecting carcass and organs for white cysts
20. You cannot diagnose it in a slaughtered pig
21. Do not know
22. What should ideally be done with a live pig with cysticercosis?
23. Treat the pig with ash
24. Give the pig medicine
25. Isolate the pig from the other pigs
26. No treatment is needed
27. Kill the pig as it is contagious
28. Sell the pig
29. Do not know
30. What can be done with a slaughtered pig with cysticercosis?
31. Wait a day before eating the pork
32. Put salt on the pork before eating
33. Do not eat the pork, but sell the pork in the local area
34. Do not eat the pork, but sell the pork to another area
35. Destroy the pig
36. Cut out the infected part and eat what is left
37. Thoroughly boil the meat before eating
38. Do not know
39. How can you prevent pigs from getting porcine cysticercosis?
40. By confining pigs and piglets, so they do not get in contact with human stool
41. Stop open defecation of all ages and use latrines with doors that can be closed
42. Only feed pigs and piglets with clean vegetables and water
43. All of the above answers are correct
44. None of the above answers are correct
45. Do not know
46. Is porcine cysticercosis a problem for human health?
47. No, pigs only infect other pigs
48. No, pigs infect other animals but humans do not get infected
49. Yes, humans can get a tapeworm by eating infected pork
50. Yes, humans can get a skin infection if direct contact with an infected pig
51. Yes, humans can get neurology symptoms by eating infected pork
52. Do not know
53. Are porcine cysticercosis and human tapeworm related?
54. No, It is caused by two different parasites
55. No, only pigs get infected and it cannot be transmitted to humans
56. Yes, but only people with epileptic seizures (fits) can transmit the disease
57. Yes, both pig and human are necessary to maintain the parasite life cycle
58. Do not know
59. What is human tapeworm infection/taeniosis?
60. It is a long worm that lives in the human’s intestine
61. It is a small worm that lives under the human’s skin
62. It is a long worm that lives in the pig’s intestine
63. It is a small worm that lives under the pig’s skin
64. It is a long worm that lives in pigs and in the environment
65. Do not know
66. What problems can an adult Taenia solium tapeworm cause?
67. The adult tapeworm cause severe stomach pain
68. The adult tapeworm can give headaches
69. The adult tapeworm can give seizures (fits)
70. There is a risk of infecting pigs and humans with cysticercosis
71. Do not know
72. How do people get Taenia solium tapeworm infection?
73. By eating raw or undercooked infected pork
74. By eating rice or maize bran
75. By drinking contaminated water
76. By physical contact with sick people
77. Humans do not get infected
78. Do not know
79. How can human tapeworm infection/taeniosis be diagnosed?
80. By looking at a faecal sample under a microscope
81. By taking a scrap from the skin and look under a microscope
82. By waiting until there is sign of headache
83. By checking the pigs for worms
84. Taeniosis cannot be diagnosed
85. Do not know
86. A person infected with a Taenia solium tapeworm will shed many tapeworm eggs through…
87. Coughing
88. Defecating
89. Urinating
90. Sharing drinks
91. None of the above
92. Do not know
93. How can taeniosis be treated?
94. By drinking green tea
95. By washing hands more often and being isolated from others
96. By treating with worm medicine
97. By going to the traditional healer
98. By only eating rice for 1 week so the worm dies
99. Do not know
100. How can you prevent human tapeworm?
101. By washing hands before eating
102. By cooking the pork so it is well done
103. By not being close to persons with neurocysticercosis
104. By not sharing drinks with sick people
105. By no physical contact with pigs
106. Do not know
107. What is human neurocysticercosis?
108. It is a disease with skin wounds caused by the virus Cysticercus
109. It is a disease with stomach cramps caused by the virus Cysticercus
110. It is a disease with cysts caused by eating mouldy maize or rice
111. It is a disease with cysts in the person’s tissues including the brain
112. Do not know
113. A person with neurocysticercosis/cysticercosis may have got the infection by?
114. Eating vegetables contaminated with tapeworm eggs from human faeces
115. Eating undercooked pork infected with *Taenia solium*
116. Physical contact with an infected pig
117. Being together with a person who has epileptic seizures (fits)
118. Do not know
119. What are the symptoms of neurocysticercosis?
120. Epileptic seizures (fits)
121. Headache
122. Dizziness
123. All of the above symptoms
124. None of the above symptoms
125. Do not know
126. What should a person who experience seizures or has chronic headache do?
127. Seek medical assistance
128. Be isolated from others
129. Not share drinks with others
130. Drink 500 ml of water and not eat meat
131. Do not know
132. A person with neurocysticercosis can transmit the disease to other people through?
133. Stool
134. Direct skin contact with other people
135. Cough
136. Sharing drinks with other
137. Neurocysticercosis cannot be transmitted to other people
138. Do not know
139. How can human cysticercosis be prevented?
140. By isolating infected pigs
141. By isolating people with neurocysticercosis
142. By no drinks sharing
143. By washing vegetable in clean water and hands with soap
144. By treating human tapeworm carriers
145. All of the above are correct
146. Do not know

**2b. Questionnaire 2 (QS2)**

1. Have you ever heard about *masese/mase/mushokwe*, or The Vicious Worm?
2. No
3. Yes, it is a disease of people and pigs
4. Yes, it is a disease of dogs and sheep
5. Yes, it is a disease of goats
6. What is porcine cysticercosis, or The Vicious Worm in pigs?
7. Disease with white cysts caused by the tapeworm *Taenia solium*
8. Disease with white cysts caused by maize bran
9. Wounds in the skin caused by thorns
10. Do not know
11. What does porcine cysticercosis look like in pigs?


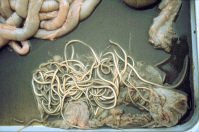


B: Worms in the intestine


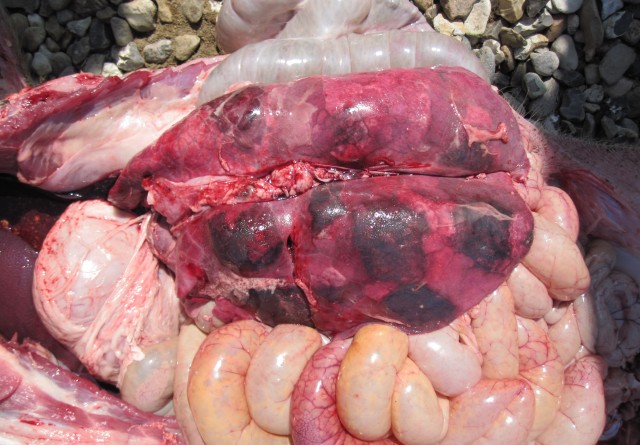


A: Black areas on the lungs


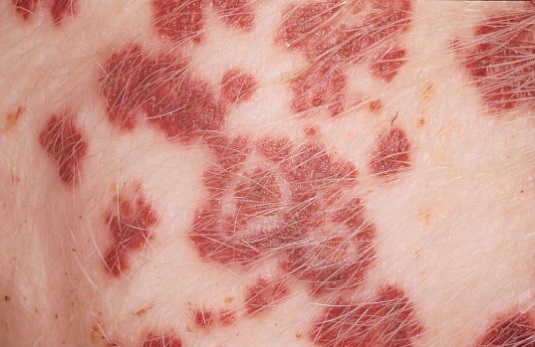


C: Red circular marks on the skin


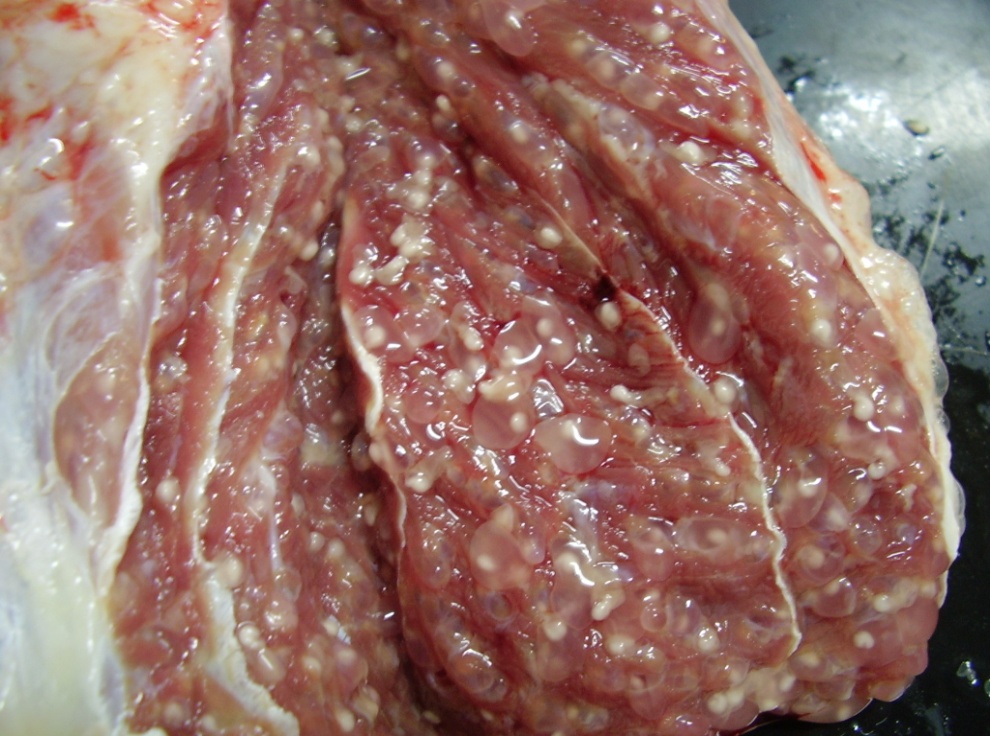


D: White cysts in the meat and organs

1. How can a pig become infected with porcine cysticercosis?
2. From dirt getting into wounds
3. From eating human stool
4. From eating mouldy maize bran or rice
5. From being mated with an infected pig
6. Can you eat the meat of a slaughtered pig that has cysticercosis?
7. Yes, because humans cannot get infected from the meat
8. Yes, but only if you put a lot of salt on the meat
9. Yes, but only after the meat is dried
10. Yes, but only if you cook the meat thoroughly before eating it, otherwise you have to throw it away
11. Can you prevent pigs from getting porcine cysticercosis?
12. No, pigs are born infected
13. Yes, by changing the person who feeds the pigs
14. Yes, by confining pigs to stop them from coming into contact with human stool
15. Yes, by not feeding pigs brewing residues
16. What is human tapeworm infection/taeniasis?
17. Infection with a long flat worm that lives in the human’s intestine, and sheds small parts in the stool
18. Infection with a long round worm that lives in the human’s intestine and is seen in the stool
19. Infection with a small round worm that lives under the human’s skin
20. Infection with a long worm that lives in pigs and in the environment
21. How do people get *Taenia solium* tapeworm infection?
22. By eating raw or undercooked infected pork
23. By direct contact with an infected pig
24. By physical contact with sick people
25. Humans do not get infected
26. How does a person with a tapeworm shed the tapeworm eggs into the environment?
27. Through droplets when they cough or sneeze
28. With their stool when they defecate
29. Through saliva when they kiss or share drinks with someone
30. Humans can not transmit the disease
31. How can human tapeworm infections/taeniasis be treated?
32. By drinking green tea
33. By going to the traditional healer
34. By taking deworming medicine from the health centre
35. By only eating rice for 1 week so the worm dies
36. How can you prevent human tapeworm infections/taeniasis?
37. By always cooking pork thoroughly before eating it
38. By avoiding physical contact with infected people
39. By avoiding physical contact with infected pigs
40. You cannot prevent human tapeworm infections
41. How do people become infected with cysticercosis?
42. By accidentally eating tapeworm eggs from human faeces from people with a tapeworm, e.g. contaminated vegetables, dirty hands
43. By eating undercooked pork infected with *Taenia solium* masese (cysticercosis)
44. From having physical contact with an infected pig with *Taenia solium* masese (cysticercosis)
45. By sharing drinks with an infected person
46. Can people with neurocysticercosis transmit the disease to others?
47. No, neurocysticercosis cannot be transmitted to other people
48. Yes, by direct skin contact
49. Yes, by coughing or sneezing
50. Yes, by sharing drinks
51. What are the symptoms of neurocysticercosis?
52. Epileptic seizures (fits), headache, dizziness
53. Stomach cramps, diarrhoea, vomiting
54. Chest pain, difficulty breathing
55. There are no symptoms in humans
56. How can human cysticercosis and neurocysticercosis be prevented?
57. By isolating infected pigs
58. By isolating infected people
59. By washing hands after defecating, and before preparing and consuming food
60. By not eating pork
